# Strengthening the primary health care for non-communicable disease prevention and control in the post-pandemic period: a perspective from China

**DOI:** 10.1186/s41256-023-00336-9

**Published:** 2023-11-29

**Authors:** Zhangyang Pan, Jing Wu, Yunguo Liu

**Affiliations:** 1 Global Health Research Center, Duke Kunshan University, Kunshan, Jiangsu China; 2 The Center for Chronic Noncommunicable Diseases Prevention and Control, China Centers for Disease Control and Prevention, Beijing, China

**Keywords:** Non-communicable diseases, Primary health care, Post-pandemic, Healthy China 2030

## Abstract

Non-communicable diseases (NCDs) have become the leading cause of deaths in China and many other countries worldwide. To call for actions in strengthening primary health care (PHC) and accelerate NCD prevention and control in the post-pandemic era in China, the 2023 Duke Kunshan Health Forum focused on innovative approaches and lessons learned during the pandemic that can be applied in addressing NCD challenges. In this article we summarize key points discussed by the participants in three areas: PHC as the foundation and ultimate solution for NCD prevention and control, post-pandemic opportunities to accelerate the NCD program with innovative approaches, and an action framework proposed by the Forum collaborators to address remaining challenges and achieve NCD control objectives in China. The core of the suggested action framework is to offer people-centered, lifetime, comprehensive, continued, and quality NCD prevention and control services, which rely on an integrated healthcare system connecting the primary, secondary, and tertiary levels of care. To achive this objective, six interconnected actions are recommended in the framework: prioritizing and integrating NCD in PHC and Universal Health Coverage (UHC) framework, engaging multiple stakeholders, directing resources to PHC for quality NCD services, leveraging advantages of new technology, encouraging the use of PHC and improving services, and strengthening best practice sharing.

## Background

The COVID-19 outbreak further disrupted the services for NCD prevention and treatment among others, triggering a potential upsurge in the NCD disease burden in many countries [1]. In the past three decades, dramatic socioeconomic changes have shaped the epidemiological and demographic landscape in China, manifesting a fast shift of disease burden from communicable diseases to NCDs. According to the 2021 Chinese Death Surveillance Dataset, NCDs accounted for 89.32% of all causes of deaths in the country [2]. To address this urging challenge, with respect to the implementation of the “Healthy China 2030” strategy and the “14th Five-Year Plan for National Health”, the Duke Kunshan Health Forum was convened in April 2023 in Duke Kunshan University, China. About 200 Chinese health policy-makers, scholars and health practioners attended the Forum, which themed “Strengthen the primary healthcare, prevent non-communicable diseases, promote health for all.” It aimed to provide a China perspective on advancing research and practice for NCD prevention and control in the post-pandemic era. Three sub-themes were discussed during the concurrent sessions, namely, epidemic trends and risk factors of NCDs, integrated health systems for NCD prevention and control, and technological innovation for NCD control and PHC strengthening. In this article we summarize the evolvement of policies and implementations exchanged in the Forum, highlight the importance of PHC in NCD control and post-pandemic opportunities to accelerate NCD program, and discuss key elements of an action framework proposed by the Forum collaborators.

## PHC as the foundation and ultimate solution for NCD prevention and control in China

NCDs are preventable and controllable. The PHC, known as the cornerstone of the health system, has been widely acknowledged as a key to NCD response. The strong political commitment and continuous efforts laid a solid foundation for tackling NCD at the PHC level in China. As part of the 2009 China health reform, the Basic Public Health Service (BPHS) program was launched for all Chinese citizens. Fully funded by the central and local governments, the service package had expanded from only 9 categories initially to 31 categories in 2022, adding more NCD-related services into its scope. Since 2009, the health financing reform also guaranteed health insurance coverage for over 95% of the Chinese population [3]. There were also a growing number of national policies being introduced for the “PHC-focused NCD prevention and control” [4]. For instance, 59 out of the 124 main indicators of the Healthy China 2030 Action Plan are related to NCDs. Reducing premature mortality caused by NCDs is also one of the critical objectives of China's 14th Five-Year Plan for National Health. In January 2023, a policy guideline published by the State Council of China further enhanced the importance of PHC strengthening by adding it to the national strategies for promoting rural revitalization.

Moreover, a series of key initiatives had been rolled out in recent years, including the National Demonstration Areas Program for Integrated NCDs Prevention and Control (NCDDA), China Healthy Lifestyle for All campaign, and the Whole-of-Care Intervention to the Prioritized NCD Population in Rural China. Many of these demonstration programs focus on the PHC capacity building and the PHC infrastructure improvement. Other ongoing health system restructuring, such as the Integrated County Healthcare Consortium, also witnessed positive results in the BPHS service provision [5].

Building upon all these efforts, China has made impressive achievements regarding PHC strengthening and HUHC. The 2023 monitoring report issued by the World Health Organization and the World Bank showed that China has achieved the UHC service coverage index over 80 since 2019 as one of the Sustainable Development Goals [6]. Future NCD prevention and control actions require continuous political commitment and the scaling up of best practices from these demonstration programs and comgaigns.

## Post-pandemic opportunities to accelerate the NCD program with innovative approaches

The COVID-19 pandemic heightened the understanding that the traditional boundaries separating communicable and non-communicable diseases were blurred. Individuals with pre-existing chronic health conditions were more vulnerable to experiencing severe illness and mortality if infected with the virus. Some successful experience of the pandemic control in China provided new insights and created an opportunity window for upgrading the NCD management, as described below.

(1) The multi-sectoral coordination between health and non-health sectors and concerted actions within the health system among the three-level facilities (primary-secondarily-tertiary) during the pandemic could be valuable practices for future NCD prevention and control. (2) Innovative approaches developed by the public and private sectors to fight the pandemic are feasible for NCD prevention and control. Digital health interventions, such as medical consultation apps and Internet hospitals, integrated health services into one platform. NCD patients could seek consultation, obtain prescriptions, track medication delivery, and receive health education without commuting to health facilities. Studies found that digital health intervention generated consistently positive results in health service effectiveness in many low- and middle-income countries, as it improved service accessibility, upgraded user experience, and expanded healthcare providers’ capacity [7]. However, limitations were also detected for the digital tools as mixed results were generated on NCD patients’ health outcome after the intervention [7]. (3) Community grid management could be modified and applied in NCD prevention and control. This new governance mode emerged at the grassroots level during the pandemic, which divided the communities into smaller grids and assigned responsibilities for quick response to epidemic with the aid of information science and broad social mobilization [8]. As NCD prevention and control requires multiple stakeholders' engagement and effective interventions, the legacies of new technology application and cross-sectoral collaboration would be leveraged in the future NCD prevention and control action plan.

## A proposed action framework to address remaining challenges and achieve NCD control objectives in China

After decades of efforts and resource inputs to accomplish the broad coverage of health services, the quality of care improvement at the PHC level remains essential to achieve NCD control objectives in the post-pandemic era. There are a few challenges that require attention from all relevant stakeholders.

(1) Given the large size of the total population, the aging trend, and increased service demand from the patients’ side, the national BPHS program may face continuous financing pressure, especially in some less developed provinces [9]. As the operation of PHC facilities primarily relies on government funding, lacking subsidies would result in less purchasing power to upgrade the equipments and cover salaries for the health workforce, which might adversely impact the service provision. (2) Insufficient capacity building and limited career development incentives would hinder health workforce retention at the PHC level [9]. Shortage in human resources may become one of the bottlenecks of high-quality care provision at the PHC level. (3) The slow progress of integration between clinical care and public health services brought unsatisfied patient experiences, which further diminished the trust in the PHC and drove people to bypass the PHC services. For instance, patients might face different doctors for every consecutive visit for NCD medication prescriptions, as well as repeat clinical checks due to the disconnection of different health information systems [9]. Thus, future actions would require continuous resource input into the grassroots levels for operation maintenance, talent retention, and service improvement.

**Fig. 1:**
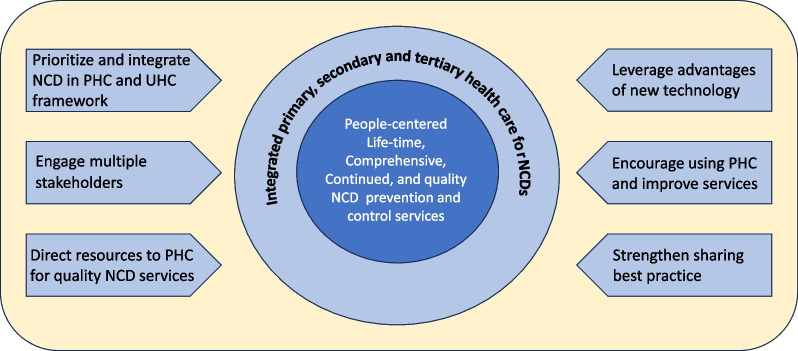
Proposed action framework to strengthen PHC for accelerating NCD prevention and control in China

With the favored policy environment and the opportunities that emerged from the pandemic, an action framework was jointly proposed during the 2023 Duke Kunshan Health Forum (Fig. 1), which aimed to drive the NCD prevention and control work to the next level.

The framework focuses on achieving a people-centered, lifetime, comprehensive, continued, and quality NCD prevention and control services. The service provision relies on a health care system that integrates the primary, secondary, and tertiary level care in a coordinative and synergetic manner. As addressed in previous sections, six interconnected actions can drive continuous improvement of the whole system (Fig. 1). NCD services for the whole country population is essential as highlighted in the WHO initiated UHC framework and PHC strengthening. Therefore, prioritizing and integrating NCDs in the PHC and the UHC framework would place the issue on a higher political agenda, which can enhance multisectoral collaboration and direct more resources to sustain and upgrade quality care provision. Besides, leveraging the advantages of new technology, encouraging mutual learning of best practices between different provinces, and localizing other countries’ successful experiences would accelerate service improvement at the grassroots [10].

## Conclusions

With decades of efforts in forging UHC, China has laid a solid PHC foundation for NCD prevention and control. However, more robust actions are needed to bring NCD prevention and control to the next level through a people-centered and integrated PHC system. The pandemic posed the urgencies and the opportunities for future interventions, which require continuous political commitment and resource inputs. The action framework the Forum proposed also called for multiple stakeholders' engagement, leveraging the advantages of new technology, encouraging patients to use PHC and improving services, and sharing best practices. Lessons learned from the ongoing demonstration programs, especially the NCDDA mode, would enrich the framework and help build up future national and local implementation plans for extending the practice in all communities across China, paving the way toward fully achieving SDG targets and Healthy China 2030 goals.

## Data Availability

Not applicable.

## Abbreviations

BPHS: Basic Public Health Service
NCDDA: National Demonstration Areas Program for Integrated NCD Prevention and Control
NCDs: Non-communicable diseases
PHC: Primary Health Care
UHC: Universal Health Coverage

## References

[1] Haileamlak A (2022). The impact of COVID-19 on non-communicable diseases. Ethiop J Health Sci.

[2] Wu J, Zhou M, Su X. Chinese Death Surveillance Dataset, 2021 [Internet]. China Science and Technology Press; 2022 [cited 2023 Nov 5]. Available from: https://ncncd.chinacdc.cn/xzzq_1/202101/W020230317353700905354.pdf

[3] Zhou Y, Li C, Wang M, Xu S, Wang L, Hu J (2022). Universal health coverage in China: a serial national cross-sectional study of surveys from 2003 to 2018. Lancet Public Health.

[4] Xiong S, Cai C, Jiang W, Ye P, Ma Y, Liu H (2023). Primary health care system responses to non-communicable disease prevention and control: a scoping review of national policies in Mainland China since the 2009 health reform. Lancet Reg Health West. Pac.

[5] Gao Q, Ma Y, Zhu P, Chen D (2022). Healthcare professionals’ views of the integrated county healthcare consortium in Zhejiang, China. Int J Integr Care.

[6] WHO. Tracking Universal Health Coverage: 2023 Global monitoring report [Internet]. World Health Organization and the International Bank for Reconstruction and Development/The World Bank; 2023 [cited 2023 Nov 7]. Available from: https://www.who.int/publications/i/item/9789240080379

[7] Xiong S, Lu H, Peoples N, Duman EK, Najarro A, Ni Z (2023). Digital health interventions for non-communicable disease management in primary health care in low-and middle-income countries. npj Digit Med.

[8] Ling C, Wen X (2020). Community grid management is an important measure to contain the spread of novel coronavirus pneumonia (COVID-19). Epidemiol Infect.

[9] Long Q, Jia Y, Li J, Lou Z, Liu Y. National Basic Public Health Services Programme in China: case study [Internet]. World Health Organization; 2023. Available from: https://pesquisa.bvsalud.org/portal/resource/pt/who-373220?lang=en

[10] OECD, World Health Organization. Purchasing for Quality Chronic Care: Summary Report [Internet]. OECD; 2023 [cited 2023 Nov 7]. Available from: https://www.oecd-ilibrary.org/social-issues-migration-health/purchasing-for-quality-chronic-care_66dfc7e1-en

