# Correction: Strengthening the primary health care for non-communicable disease prevention and control in the post-pandemic period: a perspective from China

**DOI:** 10.1186/s41256-023-00343-w

**Published:** 2023-12-18

**Authors:** Zhangyang Pan, Jing Wu, Yunguo Liu

**Affiliations:** 1 Global Health Research Center, Duke Kunshan University, Kunshan, Jiangsu China; 2 The Center for Chronic Noncommunicable Diseases Prevention and Control, China Centers for Disease Control and Prevention, Beijing, China

**Correction to: Global Health Research and Policy (2023) 8:49** 10.1186/s41256-023-00336-9

Following publication of the original article [1], it was reported that the institution numbers of Jing Wu and Yunguo Liu were incorrectly labelled. The institution number for Jing Wu should be 2 instead of 1 and the institution number for Yunguo Liu should be 1 instead of 2.

The correct institution numbers have been corrected in this Correction.

The original article [1] has been corrected.
